# Supplementary material for: HIVprotI: an integrated web based platform for prediction and design of HIV proteins inhibitors
Source: J Cheminform. 2018 Mar 9;10:12. doi: 10.1186/s13321-018-0266-y (PMC5845081; doi:10.1186/s13321-018-0266-y)
Supplement: Supplementary file 2 — Additional file 2. Source code of HIVProtI web server. [file 13321_2018_266_MOESM2_ESM.zip › HIVprotI_Source-code/hivprotI/weka-3-6-6/changelogs/CHANGELOG-3-5-8.html]

Weka-3-5-8 Changelog


# Weka-3-5-8 Changelog

Designed for use with Apache Ant.

---

2008-07-14 22:30 mhall

```
Non-dynamic is a lot faster.
```

- gui/GenericPropertiesCreator.props (1.19)

2008-07-14 02:29 mhall

```
Small changes to support environment variables
```

- core/converters/AbstractFileLoader.html (1.5)

2008-07-14 02:26 mhall

```
Small changes to support environment variables
```

- gui/beans/xml/XMLBeans.html (1.11)

2008-07-14 02:26 mhall

```
Small changes to support environment variables
```

- gui/beans/KnowledgeFlowApp.html (1.32)
- gui/beans/SerializedModelSaver.html (1.6)

2008-07-13 23:22 mhall

```
Added LatentSemanticAnalysis
```

- gui/GenericObjectEditor.props (1.121)

2008-07-07 23:14 mhall

```
branches:  1.1.2;
Initial import
```

- core/Environment.html (1.1)

2008-07-07 22:37 mhall

```
Peter's changes to allow export of EPS images and setting of background and legend border text
```

- gui/beans/Beans.props (1.15)
- gui/beans/StripChart.html (1.17)

2008-07-06 02:18 mhall

```
Initial import. Amri Napolitano's GSoC contribution: latent semantic analysis
```

- attributeSelection/LatentSemanticAnalysis.html (1.1)

2008-07-01 02:17 mhall

```
No longer uses serialization to create copies of BinarySMO. Apparantly speeds things up when the option for cross-validation is turned on.
```

- classifiers/functions/SMO.html (1.69)

2008-06-25 03:17 mhall

```
Updated for changes to clusterers
```

- filters/unsupervised/attribute/AddCluster.html (1.13)
- filters/unsupervised/attribute/ClusterMembership.html (1.16)

2008-06-25 03:16 mhall

```
Updated for changes to clusterers
```

- experiment/DensityBasedClustererSplitEvaluator.html (1.3)

2008-06-25 03:15 mhall

```
Updated for changes to clusterers
```

- classifiers/meta/ClassificationViaClustering.html (1.5)

2008-06-25 03:14 mhall

```
branches:  1.1.2;
Clusterer and DensityBasedClusterer renamed to AbstractClusterer and AbstractDensityBasedClusterer respectively; Clusterer and DensityBasedClusterer now interfaces
```

- clusterers/AbstractClusterer.html (1.1)
- clusterers/AbstractDensityBasedClusterer.html (1.1)

2008-06-25 03:14 mhall

```
Clusterer and DensityBasedClusterer renamed to AbstractClusterer and AbstractDensityBasedClusterer respectively; Clusterer and DensityBasedClusterer now interfaces
```

- clusterers/CLOPE.html (1.5)
- clusterers/CheckClusterer.html (1.11)
- clusterers/ClusterEvaluation.html (1.44)
- clusterers/Clusterer.html (1.18)
- clusterers/DBScan.html (1.9)
- clusterers/DensityBasedClusterer.html (1.9)
- clusterers/MakeDensityBasedClusterer.html (1.18)
- clusterers/OPTICS.html (1.8)
- clusterers/RandomizableClusterer.html (1.3)
- clusterers/RandomizableDensityBasedClusterer.html (1.3)
- clusterers/RandomizableSingleClustererEnhancer.html (1.3)
- clusterers/SingleClustererEnhancer.html (1.4)

2008-06-23 09:43 mhall

```
branches:  1.1.2;
Initial import
```

- attributeSelection/FilteredSubsetEval.html (1.1)

2008-06-23 09:21 mhall

```
Wrong default filter in setOptions
```

- attributeSelection/FilteredAttributeEval.html (1.2)

2008-06-22 22:16 mhall

```
Doc update
```

- attributeSelection/CostSensitiveASEvaluation.html (1.3)

2008-06-19 21:18 mhall

```
Doc fix
```

- classifiers/trees/DecisionStump.html (1.27)

2008-06-19 04:17 mhall

```
Javadoc update
```

- associations/FilteredAssociator.html (1.4)
- associations/HotSpot.html (1.6)
- attributeSelection/CheckAttributeSelection.html (1.7)
- attributeSelection/LinearForwardSelection.html (1.4)
- attributeSelection/SubsetSizeForwardSelection.html (1.4)
- classifiers/bayes/AODEsr.html (1.4)
- classifiers/bayes/DMNBtext.html (1.2)
- classifiers/bayes/NaiveBayesUpdateable.html (1.11)
- classifiers/bayes/net/BIFReader.html (1.15)
- classifiers/bayes/net/EditableBayesNet.html (1.3)
- classifiers/functions/supportVector/NormalizedPolyKernel.html (1.8)
- classifiers/functions/supportVector/PolyKernel.html (1.9)
- classifiers/functions/supportVector/RBFKernel.html (1.11)
- classifiers/functions/supportVector/StringKernel.html (1.9)
- classifiers/meta/MultiClassClassifier.html (1.48)
- classifiers/meta/RandomCommittee.html (1.13)
- classifiers/meta/ThresholdSelector.html (1.43)
- classifiers/mi/supportVector/MIPolyKernel.html (1.4)
- classifiers/mi/supportVector/MIRBFKernel.html (1.4)

2008-06-19 04:17 mhall

```
Javadoc update
```

- classifiers/rules/DTNB.html (1.4)
- classifiers/trees/FT.html (1.6)
- classifiers/trees/J48graft.html (1.3)
- clusterers/EM.html (1.44)
- clusterers/FilteredClusterer.html (1.4)
- clusterers/SimpleKMeans.html (1.39)
- core/stemmers/SnowballStemmer.html (1.10)
- core/tokenizers/NGramTokenizer.html (1.4)
- core/tokenizers/WordTokenizer.html (1.4)
- estimators/CheckEstimator.html (1.5)
- filters/supervised/attribute/AttributeSelection.html (1.9)
- filters/unsupervised/attribute/NominalToString.html (1.6)
- filters/unsupervised/attribute/StringToWordVector.html (1.25)
- filters/unsupervised/instance/ReservoirSample.html (1.4)

2008-06-19 01:19 fracpete

```
added unicode support
fixed parsing of strings (crapped out when encountering numbers within strings) using a different state in the lexer
```

- filters/unsupervised/instance/subsetbyexpression/Parser.html (1.3)
- filters/unsupervised/instance/subsetbyexpression/Scanner.html (1.3)
- filters/unsupervised/instance/subsetbyexpression/Scanner.jflex (1.2)
- filters/unsupervised/instance/subsetbyexpression/sym.html (1.3)

2008-06-18 02:10 mhall

```
Doc update
```

- classifiers/Evaluation.html (1.88)

2008-06-16 22:45 mhall

```
Doc fix
```

- core/xml/XStream.html (1.4)

2008-06-16 01:19 mhall

```
Changed so that failures on nominal attribute/class now report failure due to multi-valued nominal attribute/class
```

- core/Capabilities.html (1.27)

2008-06-13 02:25 mhall

```
Made safe for execution in a headless environment.
```

- gui/beans/AttributeSummarizer.html (1.10)
- gui/beans/DataVisualizer.html (1.13)
- gui/beans/GraphViewer.html (1.9)
- gui/beans/ModelPerformanceChart.html (1.8)
- gui/beans/ScatterPlotMatrix.html (1.9)

2008-06-12 23:07 mhall

```
Fix for partitionOptions by Ingo Mierswa - allows additional options to the search to be given after the -- without having to explicitly specify the estimator with -E
```

- classifiers/bayes/BayesNet.html (1.33)

2008-06-11 04:24 mhall

```
Fixed null ptr exception in incremental loading. Added some progress output for incremental loading.
```

- gui/beans/Loader.html (1.23)

2008-06-09 03:22 mhall

```
Improved stopping.
```

- gui/beans/CrossValidationFoldMaker.html (1.13)
- gui/beans/PredictionAppender.html (1.19)
- gui/beans/Saver.html (1.8)
- gui/beans/TestSetMaker.html (1.8)
- gui/beans/TrainTestSplitMaker.html (1.14)
- gui/beans/TrainingSetMaker.html (1.8)

2008-06-09 02:44 mhall

```
Added a method for stopping all flows. If the thread that the FlowRunner is running in gets interrupted, then all flows get stopped
```

- gui/beans/FlowRunner.html (1.9)

2008-06-09 02:44 mhall

```
Removed some debugging output
```

- gui/beans/Associator.html (1.8)
- gui/beans/Classifier.html (1.33)
- gui/beans/Clusterer.html (1.10)
- gui/beans/Filter.html (1.20)

2008-06-05 23:51 mhall

```
Fixed formating of coefficients/odds ratio column
```

- classifiers/functions/Logistic.html (1.42)

2008-06-05 23:48 mhall

```
Fixed formatting of priors in toString()
```

- classifiers/bayes/NaiveBayes.html (1.27)

2008-05-29 02:23 mhall

```
Added support for GraphListeners
```

- gui/beans/AssociatorBeanInfo.html (1.3)

2008-05-20 22:36 mhall

```
Fixed bug in the handling of nominal values with the 'is' operator when missing values are present
```

- filters/unsupervised/instance/subsetbyexpression/Parser.cup (1.2)

2008-05-19 04:21 mhall

```
Fixed parameter rounding (now uses Math.rint() so that, for example, -1 is not rounded to 0.
```

- classifiers/meta/CVParameterSelection.html (1.37)

2008-05-15 04:14 mhall

```
Fixed bug where class-distribution-spreads < 1 caused the minority class to be undersampled as well as the other classes.
```

- filters/supervised/instance/SpreadSubsample.html (1.9)

2008-05-15 02:16 fracpete

```
added filter for selecting subsets of a dataset, based on CUP and JFlex
fixed "=" in MathematicalExpression parser
simplified parser/lexer generation in parsers.xml ANT build file
```

- parsers.xml (1.2)
- core/MathematicalExpression.html (1.8)
- core/mathematicalexpression/Parser.cup (1.2)
- core/mathematicalexpression/Parser.html (1.2)
- core/mathematicalexpression/Scanner.html (1.2)
- core/mathematicalexpression/Scanner.jflex (1.2)
- core/mathematicalexpression/sym.html (1.2)
- filters/unsupervised/instance/SubsetByExpression.html (1.1)

2008-05-13 04:10 mhall

```
Now uses the current class loader rather than the system class loader to load icons
```

- core/Version.html (1.8)

2008-05-13 04:01 mhall

```
Now uses the current class loader rather than the system class loader to load icons
```

- gui/beans/BeanVisual.html (1.10)

2008-05-13 03:58 mhall

```
Updated year to 2008
```

- core/Copyright.props (1.2)

2008-05-13 03:57 mhall

```
Now uses the current class loader rather than the system class loader
```

- core/Copyright.html (1.2)

2008-05-12 01:01 cvs\_rrb

```
Enable datagenerator properly
```

- classifiers/bayes/net/GUI.html (1.2)

2008-05-09 09:14 fracpete

```
rewrite of weka.core.MathematicalExpression using JFlex and CUP parser generators:
http://jflex.de/
http://www2.cs.tum.edu/projects/cup/
```

- build.xml (1.44)
- core/parser/JFlex/Action.html (1.1)
- core/parser/JFlex/CharClassException.html (1.1)
- core/parser/JFlex/CharClassInterval.html (1.1)
- core/parser/JFlex/CharClasses.html (1.1)
- core/parser/JFlex/CharSet.html (1.1)
- core/parser/JFlex/CharSetEnumerator.html (1.1)
- core/parser/JFlex/DFA.html (1.1)
- core/parser/JFlex/EOFActions.html (1.1)
- core/parser/JFlex/Emitter.html (1.1)
- core/parser/JFlex/ErrorMessages.html (1.1)
- core/parser/JFlex/IntCharSet.html (1.1)
- core/parser/JFlex/IntPair.html (1.1)
- core/parser/JFlex/Interval.html (1.1)
- core/parser/JFlex/LexParse.cup (1.1)
- core/parser/JFlex/LexScan.html (1.1)
- core/parser/JFlex/LexicalStates.html (1.1)
- core/parser/JFlex/Macros.html (1.1)
- core/parser/JFlex/Main.html (1.1)
- core/parser/JFlex/Messages.properties (1.1)
- core/parser/JFlex/NFA.html (1.1)
- core/parser/JFlex/RegExp2.html (1.1)
- core/parser/JFlex/ScannerException.html (1.1)
- core/parser/JFlex/SemCheck.html (1.1)
- core/parser/JFlex/Skeleton.html (1.1)
- core/parser/JFlex/StatePairList.html (1.1)
- core/parser/JFlex/StateSetEnumerator.html (1.1)
- core/parser/JFlex/StdOutWriter.html (1.1)
- core/parser/JFlex/Timer.html (1.1)
- core/parser/JFlex/sym.html (1.1)
- core/parser/java\_cup/runtime/ComplexSymbolFactory.html (1.1)
- core/parser/java\_cup/runtime/DefaultSymbolFactory.html (1.1)
- core/parser/java\_cup/runtime/Scanner.html (1.1)
- core/parser/java\_cup/runtime/Symbol.html (1.1)
- core/parser/java\_cup/runtime/SymbolFactory.html (1.1)
- core/parser/java\_cup/runtime/lr\_parser.html (1.1)
- core/parser/java\_cup/runtime/virtual\_parse\_stack.html (1.1)
- filters/unsupervised/attribute/MathExpression.html (1.10)

2008-05-09 09:14 fracpete

```
rewrite of weka.core.MathematicalExpression using JFlex and CUP parser generators:
http://jflex.de/
http://www2.cs.tum.edu/projects/cup/
```

- core/parser/JFlex/CountEmitter.html (1.1)
- core/parser/JFlex/GeneratorException.html (1.1)
- core/parser/JFlex/HiLowEmitter.html (1.1)
- core/parser/JFlex/LexParse.html (1.1)
- core/parser/JFlex/LexScan.flex (1.1)
- core/parser/JFlex/MacroException.html (1.1)
- core/parser/JFlex/Options.html (1.1)
- core/parser/JFlex/Out.html (1.1)
- core/parser/JFlex/PackEmitter.html (1.1)
- core/parser/JFlex/RegExp.html (1.1)
- core/parser/JFlex/RegExp1.html (1.1)
- core/parser/JFlex/RegExps.html (1.1)
- core/parser/JFlex/SilentExit.html (1.1)
- core/parser/JFlex/StateSet.html (1.1)
- core/parser/JFlex/skeleton.default (1.1)
- core/parser/java\_cup/ErrorManager.html (1.1)
- core/parser/java\_cup/Lexer.html (1.1)
- core/parser/java\_cup/Lexer.jflex (1.1)
- core/parser/java\_cup/Main.html (1.1)
- core/parser/java\_cup/action\_part.html (1.1)
- core/parser/java\_cup/action\_production.html (1.1)
- core/parser/java\_cup/assoc.html (1.1)
- core/parser/java\_cup/emit.html (1.1)
- core/parser/java\_cup/internal\_error.html (1.1)
- core/parser/java\_cup/lalr\_item.html (1.1)
- core/parser/java\_cup/lalr\_item\_set.html (1.1)
- core/parser/java\_cup/lalr\_state.html (1.1)
- core/parser/java\_cup/lalr\_transition.html (1.1)
- core/parser/java\_cup/lr\_item\_core.html (1.1)
- core/parser/java\_cup/non\_terminal.html (1.1)
- core/parser/java\_cup/nonassoc\_action.html (1.1)
- core/parser/java\_cup/parse\_action.html (1.1)
- core/parser/java\_cup/parse\_action\_row.html (1.1)
- core/parser/java\_cup/parse\_action\_table.html (1.1)
- core/parser/java\_cup/parse\_reduce\_row.html (1.1)
- core/parser/java\_cup/parse\_reduce\_table.html (1.1)
- core/parser/java\_cup/parser.cup (1.1)
- core/parser/java\_cup/parser.html (1.1)
- core/parser/java\_cup/production.html (1.1)
- core/parser/java\_cup/production\_part.html (1.1)
- core/parser/java\_cup/reduce\_action.html (1.1)
- core/parser/java\_cup/shift\_action.html (1.1)
- core/parser/java\_cup/sym.html (1.1)
- core/parser/java\_cup/symbol.html (1.1)
- core/parser/java\_cup/symbol\_part.html (1.1)
- core/parser/java\_cup/symbol\_set.html (1.1)
- core/parser/java\_cup/terminal.html (1.1)
- core/parser/java\_cup/terminal\_set.html (1.1)
- core/parser/java\_cup/version.html (1.1)

2008-05-05 04:10 mhall

```
Associator renamed to AbstractAssociator; Associator has turned into an interface
```

- associations/Apriori.html (1.31)
- associations/Associator.html (1.11)
- associations/AssociatorEvaluation.html (1.5)
- associations/CheckAssociator.html (1.7)
- associations/GeneralizedSequentialPatterns.html (1.3)
- associations/PredictiveApriori.html (1.14)
- associations/SingleAssociatorEnhancer.html (1.2)
- associations/Tertius.html (1.10)

2008-05-05 04:10 mhall

```
branches:  1.1.2;
Associator renamed to AbstractAssociator; Associator has turned into an interface
```

- associations/AbstractAssociator.html (1.1)

2008-04-29 03:07 mhall

```
Added another constructor
```

- gui/beans/TestSetEvent.html (1.5)

2008-04-29 03:04 mhall

```
Added another constructor
```

- gui/beans/TrainingSetEvent.html (1.5)

2008-04-23 04:53 mhall

```
Added Arne Muller's methods to shuffle attribute values
```

- core/Instance.html (1.28)
- core/Instances.html (1.76)
- core/SparseInstance.html (1.23)

2008-04-21 23:39 mhall

```
Removed a couple of infinite recursive loops.
```

- gui/CheckBoxList.html (1.3)

2008-04-21 04:26 mhall

```
Changed a & to &&
```

- core/converters/ArffLoader.html (1.23)

2008-04-20 02:47 mhall

```
Initial import
```

- classifiers/functions/supportVector/PrecomputedKernelMatrixKernel.html (1.1)

2008-04-17 05:55 mhall

```
branches:  1.2.2;
Updated javadocs.
```

- attributeSelection/CostSensitiveAttributeEval.html (1.2)
- attributeSelection/CostSensitiveSubsetEval.html (1.2)

2008-04-16 22:11 fracpete

```
branches:  1.2.2;
introduction of interface RevisionHandler which enables classes to return their CVS/Subversion revision string
```

- classifiers/bayes/net/MarginCalculator.html (1.2)

2008-04-16 22:11 fracpete

```
introduction of interface RevisionHandler which enables classes to return their CVS/Subversion revision string
```

- associations/tertius/AttributeValueLiteral.html (1.6)
- associations/tertius/Body.html (1.6)
- associations/tertius/Head.html (1.6)
- associations/tertius/IndividualInstance.html (1.5)
- associations/tertius/IndividualInstances.html (1.6)
- associations/tertius/IndividualLiteral.html (1.5)
- associations/tertius/Literal.html (1.5)
- associations/tertius/LiteralSet.html (1.7)
- associations/tertius/Predicate.html (1.5)
- associations/tertius/Rule.html (1.7)
- associations/tertius/SimpleLinkedList.html (1.6)
- classifiers/bayes/net/search/ci/CISearchAlgorithm.html (1.7)
- classifiers/bayes/net/search/ci/ICSSearchAlgorithm.html (1.8)
- classifiers/functions/neural/LinearUnit.html (1.7)
- classifiers/functions/neural/NeuralConnection.html (1.7)
- classifiers/functions/pace/ChisqMixture.html (1.5)
- classifiers/functions/pace/DiscreteFunction.html (1.4)
- classifiers/functions/pace/MixtureDistribution.html (1.5)
- classifiers/functions/pace/NormalMixture.html (1.5)
- classifiers/functions/pace/PaceMatrix.html (1.6)
- classifiers/trees/adtree/PredictionNode.html (1.7)
- classifiers/trees/adtree/ReferenceInstances.html (1.5)
- classifiers/trees/adtree/Splitter.html (1.5)
- classifiers/trees/adtree/TwoWayNominalSplit.html (1.6)
- classifiers/trees/adtree/TwoWayNumericSplit.html (1.6)
- core/neighboursearch/BallTree.html (1.2)
- core/neighboursearch/CoverTree.html (1.4)
- core/neighboursearch/KDTree.html (1.3)
- core/neighboursearch/LinearNNSearch.html (1.2)
- core/neighboursearch/NearestNeighbourSearch.html (1.2)
- core/neighboursearch/PerformanceStats.html (1.2)
- core/neighboursearch/TreePerformanceStats.html (1.2)
- core/neighboursearch/covertrees/Stack.html (1.3)
- filters/supervised/attribute/AddClassification.html (1.4)
- filters/supervised/attribute/ClassOrder.html (1.9)
- filters/supervised/attribute/Discretize.html (1.8)
- filters/supervised/instance/Resample.html (1.13)
- filters/supervised/instance/StratifiedRemoveFolds.html (1.7)

2008-04-16 22:11 fracpete

```
introduction of interface RevisionHandler which enables classes to return their CVS/Subversion revision string
```

- attributeSelection/ASEvaluation.html (1.15)
- attributeSelection/ASSearch.html (1.12)
- attributeSelection/AttributeSelection.html (1.47)
- attributeSelection/AttributeTransformer.html (1.9)
- attributeSelection/BestFirst.html (1.29)
- attributeSelection/CfsSubsetEval.html (1.28)
- attributeSelection/ChiSquaredAttributeEval.html (1.16)
- attributeSelection/ConsistencySubsetEval.html (1.17)
- attributeSelection/ExhaustiveSearch.html (1.15)
- attributeSelection/FCBFSearch.html (1.7)
- attributeSelection/GainRatioAttributeEval.html (1.23)
- attributeSelection/GeneticSearch.html (1.19)
- attributeSelection/GreedyStepwise.html (1.10)
- attributeSelection/InfoGainAttributeEval.html (1.22)
- attributeSelection/LFSMethods.html (1.3)
- attributeSelection/OneRAttributeEval.html (1.21)
- attributeSelection/PrincipalComponents.html (1.36)
- attributeSelection/RaceSearch.html (1.26)
- attributeSelection/Ranker.html (1.26)
- attributeSelection/ReliefFAttributeEval.html (1.23)
- attributeSelection/SymmetricalUncertAttributeSetEval.html (1.7)
- classifiers/bayes/blr/GaussianPriorImpl.html (1.2)
- classifiers/bayes/blr/LaplacePriorImpl.html (1.2)
- classifiers/bayes/blr/Prior.html (1.2)
- clusterers/forOPTICSAndDBScan/Utils/EpsilonRange\_ListElement.html (1.3)
- clusterers/forOPTICSAndDBScan/Utils/PriorityQueue.html (1.3)
- clusterers/forOPTICSAndDBScan/Utils/PriorityQueueElement.html (1.3)
- clusterers/forOPTICSAndDBScan/Utils/UpdateQueue.html (1.3)
- clusterers/forOPTICSAndDBScan/Utils/UpdateQueueElement.html (1.3)

2008-04-16 22:11 fracpete

```
introduction of interface RevisionHandler which enables classes to return their CVS/Subversion revision string
```

- classifiers/bayes/net/estimate/BMAEstimator.html (1.8)
- classifiers/bayes/net/estimate/BayesNetEstimator.html (1.4)
- classifiers/bayes/net/estimate/DiscreteEstimatorBayes.html (1.7)
- classifiers/bayes/net/estimate/DiscreteEstimatorFullBayes.html (1.3)
- classifiers/bayes/net/estimate/MultiNomialBMAEstimator.html (1.8)
- classifiers/bayes/net/estimate/SimpleEstimator.html (1.6)
- classifiers/lazy/kstar/KStarCache.html (1.11)
- classifiers/lazy/kstar/KStarNominalAttribute.html (1.7)
- classifiers/lazy/kstar/KStarNumericAttribute.html (1.7)
- classifiers/lazy/kstar/KStarWrapper.html (1.7)
- classifiers/misc/FLR.html (1.10)
- classifiers/misc/HyperPipes.html (1.21)
- classifiers/misc/MinMaxExtension.html (1.2)
- classifiers/misc/OLM.html (1.2)
- classifiers/misc/OSDL.html (1.2)
- classifiers/misc/SerializedClassifier.html (1.4)
- classifiers/misc/VFI.html (1.15)
- classifiers/misc/monotone/AbsoluteLossFunction.html (1.2)
- classifiers/misc/monotone/BooleanBitMatrix.html (1.2)
- classifiers/misc/monotone/Coordinates.html (1.2)
- classifiers/misc/monotone/CumulativeDiscreteDistribution.html (1.2)
- classifiers/misc/monotone/DiscreteDistribution.html (1.2)
- classifiers/misc/monotone/DistributionUtils.html (1.2)
- classifiers/misc/monotone/EnumerationIterator.html (1.2)
- classifiers/misc/monotone/InstancesComparator.html (1.2)
- classifiers/misc/monotone/InstancesUtil.html (1.3)
- classifiers/misc/monotone/MultiDimensionalSort.html (1.2)
- classifiers/misc/monotone/ZeroOneLossFunction.html (1.2)
- classifiers/trees/ft/FTInnerNode.html (1.4)
- classifiers/trees/ft/FTLeavesNode.html (1.4)
- classifiers/trees/ft/FTNode.html (1.4)
- classifiers/trees/ft/FTtree.html (1.4)
- classifiers/trees/lmt/LMTNode.html (1.8)
- classifiers/trees/lmt/LogisticBase.html (1.9)
- classifiers/trees/lmt/ResidualModelSelection.html (1.4)
- classifiers/trees/lmt/ResidualSplit.html (1.4)
- datagenerators/ClusterDefinition.html (1.5)
- datagenerators/DataGenerator.html (1.7)
- datagenerators/Test.html (1.5)
- filters/supervised/attribute/NominalToBinary.html (1.8)
- filters/supervised/attribute/PLSFilter.html (1.4)

2008-04-16 22:11 fracpete

```
introduction of interface RevisionHandler which enables classes to return their CVS/Subversion revision string
```

- classifiers/rules/ConjunctiveRule.html (1.16)
- classifiers/rules/DecisionTable.html (1.46)
- classifiers/rules/DecisionTableHashKey.html (1.3)
- classifiers/rules/JRip.html (1.22)
- classifiers/rules/M5Rules.html (1.11)
- classifiers/rules/NNge.html (1.7)
- classifiers/rules/OneR.html (1.26)
- classifiers/rules/Prism.html (1.23)
- classifiers/rules/Ridor.html (1.21)
- classifiers/rules/Rule.html (1.8)
- classifiers/rules/RuleStats.html (1.7)
- classifiers/rules/ZeroR.html (1.19)
- datagenerators/classifiers/classification/Agrawal.html (1.6)
- datagenerators/classifiers/classification/BayesNet.html (1.4)
- datagenerators/classifiers/classification/LED24.html (1.6)
- datagenerators/classifiers/classification/RDG1.html (1.5)
- filters/unsupervised/instance/NonSparseToSparse.html (1.5)
- filters/unsupervised/instance/Normalize.html (1.6)
- filters/unsupervised/instance/Randomize.html (1.7)
- filters/unsupervised/instance/RemoveFolds.html (1.6)
- filters/unsupervised/instance/RemoveFrequentValues.html (1.6)
- filters/unsupervised/instance/RemoveMisclassified.html (1.8)
- filters/unsupervised/instance/RemovePercentage.html (1.8)
- filters/unsupervised/instance/RemoveRange.html (1.8)
- filters/unsupervised/instance/RemoveWithValues.html (1.13)
- filters/unsupervised/instance/Resample.html (1.10)
- filters/unsupervised/instance/SparseToNonSparse.html (1.6)

2008-04-16 22:11 fracpete

```
introduction of interface RevisionHandler which enables classes to return their CVS/Subversion revision string
```

- classifiers/bayes/net/ADNode.html (1.7)
- classifiers/bayes/net/BayesNetGenerator.html (1.14)
- classifiers/bayes/net/ParentSet.html (1.8)
- classifiers/bayes/net/VaryNode.html (1.6)
- classifiers/bayes/net/search/local/GeneticSearch.html (1.5)
- classifiers/bayes/net/search/local/HillClimber.html (1.9)
- classifiers/bayes/net/search/local/K2.html (1.8)
- classifiers/bayes/net/search/local/LAGDHillClimber.html (1.7)
- classifiers/bayes/net/search/local/LocalScoreSearchAlgorithm.html (1.8)
- classifiers/bayes/net/search/local/RepeatedHillClimber.html (1.6)
- classifiers/bayes/net/search/local/SimulatedAnnealing.html (1.6)
- classifiers/bayes/net/search/local/TAN.html (1.7)
- classifiers/bayes/net/search/local/TabuSearch.html (1.5)
- classifiers/functions/supportVector/CheckKernel.html (1.3)
- classifiers/functions/supportVector/Kernel.html (1.8)
- classifiers/functions/supportVector/KernelEvaluation.html (1.3)
- classifiers/functions/supportVector/Puk.html (1.4)
- classifiers/functions/supportVector/RegOptimizer.html (1.3)
- classifiers/functions/supportVector/RegSMO.html (1.4)
- classifiers/functions/supportVector/RegSMOImproved.html (1.4)
- classifiers/functions/supportVector/SMOset.html (1.5)
- core/stemmers/IteratedLovinsStemmer.html (1.7)
- core/stemmers/NullStemmer.html (1.5)
- core/stemmers/Stemmer.html (1.3)
- core/stemmers/Stemming.html (1.3)

2008-04-16 22:11 fracpete

```
introduction of interface RevisionHandler which enables classes to return their CVS/Subversion revision string
```

- classifiers/evaluation/ConfusionMatrix.html (1.9)
- classifiers/evaluation/CostCurve.html (1.9)
- classifiers/evaluation/MarginCurve.html (1.11)
- classifiers/evaluation/NominalPrediction.html (1.12)
- classifiers/evaluation/NumericPrediction.html (1.9)
- classifiers/evaluation/TwoClassStats.html (1.9)
- classifiers/meta/AdaBoostM1.html (1.40)
- classifiers/meta/AttributeSelectedClassifier.html (1.26)
- classifiers/meta/Bagging.html (1.41)
- classifiers/meta/Dagging.html (1.7)
- classifiers/meta/Decorate.html (1.9)
- classifiers/meta/FilteredClassifier.html (1.28)
- classifiers/meta/LogitBoost.html (1.43)
- classifiers/meta/MetaCost.html (1.24)
- classifiers/meta/MultiBoostAB.html (1.16)
- classifiers/meta/MultiScheme.html (1.25)
- classifiers/meta/RandomSubSpace.html (1.4)
- classifiers/trees/j48/BinC45ModelSelection.html (1.11)
- classifiers/trees/j48/BinC45Split.html (1.14)
- classifiers/trees/j48/C45ModelSelection.html (1.11)
- classifiers/trees/j48/C45PruneableClassifierTree.html (1.15)
- classifiers/trees/j48/C45PruneableClassifierTreeG.html (1.2)
- classifiers/trees/j48/C45Split.html (1.13)
- classifiers/trees/j48/ClassifierSplitModel.html (1.11)
- classifiers/trees/j48/ClassifierTree.html (1.22)
- classifiers/trees/j48/Distribution.html (1.12)
- classifiers/trees/j48/EntropyBasedSplitCrit.html (1.8)
- classifiers/trees/j48/EntropySplitCrit.html (1.8)
- classifiers/trees/j48/GainRatioSplitCrit.html (1.8)
- classifiers/trees/j48/GraftSplit.html (1.2)
- classifiers/trees/j48/InfoGainSplitCrit.html (1.10)
- classifiers/trees/j48/ModelSelection.html (1.8)
- classifiers/trees/j48/NBTreeClassifierTree.html (1.6)
- classifiers/trees/j48/NBTreeModelSelection.html (1.5)
- classifiers/trees/j48/NBTreeNoSplit.html (1.4)
- classifiers/trees/j48/NBTreeSplit.html (1.5)
- classifiers/trees/j48/NoSplit.html (1.9)
- classifiers/trees/j48/PruneableClassifierTree.html (1.12)
- classifiers/trees/j48/SplitCriterion.html (1.8)
- classifiers/trees/j48/Stats.html (1.9)

2008-04-16 22:11 fracpete

```
introduction of interface RevisionHandler which enables classes to return their CVS/Subversion revision string
```

- attributeSelection/ClassifierSubsetEval.html (1.18)
- attributeSelection/RandomSearch.html (1.18)
- attributeSelection/RankSearch.html (1.19)
- attributeSelection/SVMAttributeEval.html (1.28)
- attributeSelection/SymmetricalUncertAttributeEval.html (1.22)
- attributeSelection/WrapperSubsetEval.html (1.31)
- classifiers/meta/nestedDichotomies/ClassBalancedND.html (1.8)
- classifiers/meta/nestedDichotomies/DataNearBalancedND.html (1.8)
- classifiers/meta/nestedDichotomies/ND.html (1.9)
- clusterers/Cobweb.html (1.25)
- clusterers/FarthestFirst.html (1.9)
- clusterers/XMeans.html (1.24)
- clusterers/sIB.html (1.2)
- clusterers/forOPTICSAndDBScan/Databases/SequentialDatabase.html (1.4)

2008-04-16 22:11 fracpete

```
introduction of interface RevisionHandler which enables classes to return their CVS/Subversion revision string
```

- classifiers/BVDecompose.html (1.15)
- classifiers/BVDecomposeSegCVSub.html (1.7)
- classifiers/CheckClassifier.html (1.33)
- classifiers/CheckSource.html (1.4)
- classifiers/Classifier.html (1.18)
- classifiers/CostMatrix.html (1.17)
- classifiers/EnsembleLibrary.html (1.2)
- classifiers/EnsembleLibraryModel.html (1.2)
- classifiers/EnsembleLibraryModelComparator.html (1.2)
- classifiers/JythonClassifier.html (1.2)
- classifiers/rules/PART.html (1.10)
- core/matrix/CholeskyDecomposition.html (1.5)
- core/matrix/DoubleVector.html (1.4)
- core/matrix/EigenvalueDecomposition.html (1.4)
- core/matrix/ExponentialFormat.html (1.4)
- core/matrix/FlexibleDecimalFormat.html (1.4)
- core/matrix/FloatingPointFormat.html (1.4)
- core/matrix/IntVector.html (1.4)
- core/matrix/LUDecomposition.html (1.4)
- core/matrix/LinearRegression.html (1.4)
- core/matrix/Maths.html (1.3)
- core/matrix/Matrix.html (1.8)
- core/matrix/QRDecomposition.html (1.4)
- core/matrix/SingularValueDecomposition.html (1.5)
- estimators/ConditionalEstimator.html (1.7)
- estimators/DNConditionalEstimator.html (1.8)
- estimators/Estimator.html (1.10)
- estimators/EstimatorUtils.html (1.4)
- estimators/KKConditionalEstimator.html (1.8)
- estimators/KernelEstimator.html (1.9)
- estimators/MahalanobisEstimator.html (1.8)
- estimators/NormalEstimator.html (1.10)
- estimators/PoissonEstimator.html (1.8)

2008-04-16 22:11 fracpete

```
introduction of interface RevisionHandler which enables classes to return their CVS/Subversion revision string
```

- classifiers/bayes/AODE.html (1.19)
- classifiers/bayes/BayesianLogisticRegression.html (1.3)
- classifiers/bayes/ComplementNaiveBayes.html (1.9)
- classifiers/bayes/HNB.html (1.9)
- classifiers/bayes/NaiveBayesMultinomial.html (1.16)
- classifiers/bayes/NaiveBayesMultinomialUpdateable.html (1.3)
- classifiers/bayes/NaiveBayesSimple.html (1.20)
- classifiers/bayes/WAODE.html (1.3)
- clusterers/forOPTICSAndDBScan/OPTICS\_GUI/GraphPanel.html (1.4)
- clusterers/forOPTICSAndDBScan/OPTICS\_GUI/OPTICS\_Visualizer.html (1.4)
- clusterers/forOPTICSAndDBScan/OPTICS\_GUI/ResultVectorTableModel.html (1.4)
- clusterers/forOPTICSAndDBScan/OPTICS\_GUI/SERFileFilter.html (1.3)
- clusterers/forOPTICSAndDBScan/OPTICS\_GUI/SERObject.html (1.4)
- core/neighboursearch/kdtrees/KDTreeNode.html (1.2)
- core/neighboursearch/kdtrees/KDTreeNodeSplitter.html (1.2)
- core/neighboursearch/kdtrees/KMeansInpiredMethod.html (1.2)
- core/neighboursearch/kdtrees/MedianOfWidestDimension.html (1.2)
- core/neighboursearch/kdtrees/MidPointOfWidestDimension.html (1.2)
- core/neighboursearch/kdtrees/SlidingMidPointOfWidestSide.html (1.3)
- core/stemmers/LovinsStemmer.html (1.7)
- core/xml/KOML.html (1.4)
- core/xml/MethodHandler.html (1.3)
- core/xml/PropertyHandler.html (1.4)
- core/xml/SerialUIDChanger.html (1.3)
- core/xml/XMLBasicSerialization.html (1.6)
- core/xml/XMLDocument.html (1.9)
- core/xml/XMLInstances.html (1.4)
- core/xml/XMLOptions.html (1.4)
- core/xml/XMLSerialization.html (1.16)
- core/xml/XMLSerializationMethodHandler.html (1.5)
- datagenerators/classifiers/classification/RandomRBF.html (1.4)

2008-04-16 22:11 fracpete

```
introduction of interface RevisionHandler which enables classes to return their CVS/Subversion revision string
```

- classifiers/bayes/net/search/global/GeneticSearch.html (1.5)
- classifiers/bayes/net/search/global/GlobalScoreSearchAlgorithm.html (1.10)
- classifiers/bayes/net/search/global/HillClimber.html (1.9)
- classifiers/bayes/net/search/global/K2.html (1.8)
- classifiers/bayes/net/search/global/RepeatedHillClimber.html (1.6)
- classifiers/bayes/net/search/global/SimulatedAnnealing.html (1.6)
- classifiers/bayes/net/search/global/TAN.html (1.7)
- classifiers/bayes/net/search/global/TabuSearch.html (1.5)
- classifiers/meta/AdditiveRegression.html (1.25)
- classifiers/meta/ClassificationViaRegression.html (1.27)
- classifiers/meta/CostSensitiveClassifier.html (1.29)
- classifiers/meta/END.html (1.8)
- classifiers/meta/EnsembleSelection.html (1.4)
- classifiers/meta/Grading.html (1.13)
- classifiers/meta/GridSearch.html (1.9)
- classifiers/meta/OrdinalClassClassifier.html (1.18)
- classifiers/meta/RacedIncrementalLogitBoost.html (1.12)
- classifiers/meta/RegressionByDiscretization.html (1.37)
- classifiers/meta/Stacking.html (1.32)
- classifiers/meta/StackingC.html (1.15)
- classifiers/meta/Vote.html (1.19)
- estimators/DDConditionalEstimator.html (1.8)
- estimators/DKConditionalEstimator.html (1.8)
- estimators/DiscreteEstimator.html (1.10)
- estimators/KDConditionalEstimator.html (1.8)
- estimators/NDConditionalEstimator.html (1.7)
- estimators/NNConditionalEstimator.html (1.8)

2008-04-16 22:11 fracpete

```
branches:  1.1.2;
introduction of interface RevisionHandler which enables classes to return their CVS/Subversion revision string
```

- core/RevisionUtils.html (1.1)

2008-04-16 22:11 fracpete

```
introduction of interface RevisionHandler which enables classes to return their CVS/Subversion revision string
```

- core/AlgVector.html (1.10)
- core/CheckGOE.html (1.4)
- core/ClassloaderUtil.html (1.2)
- core/GlobalInfoJavadoc.html (1.7)
- core/NormalizableDistance.html (1.2)
- core/Optimization.html (1.9)
- core/RelationalLocator.html (1.3)
- core/Statistics.html (1.10)
- filters/unsupervised/attribute/Add.html (1.7)
- filters/unsupervised/attribute/AddExpression.html (1.8)
- filters/unsupervised/attribute/AddID.html (1.6)
- filters/unsupervised/attribute/AddNoise.html (1.9)
- filters/unsupervised/attribute/Center.html (1.5)
- filters/unsupervised/attribute/ChangeDateFormat.html (1.7)
- filters/unsupervised/attribute/ClassAssigner.html (1.3)
- filters/unsupervised/attribute/Discretize.html (1.13)
- filters/unsupervised/attribute/FirstOrder.html (1.9)
- filters/unsupervised/attribute/KernelFilter.html (1.2)
- filters/unsupervised/attribute/MakeIndicator.html (1.6)
- filters/unsupervised/attribute/MultiInstanceToPropositional.html (1.7)
- filters/unsupervised/attribute/NominalToBinary.html (1.12)
- filters/unsupervised/attribute/NumericToBinary.html (1.8)
- filters/unsupervised/attribute/NumericToNominal.html (1.3)
- filters/unsupervised/attribute/PKIDiscretize.html (1.9)
- filters/unsupervised/attribute/PrincipalComponents.html (1.4)
- filters/unsupervised/attribute/PropositionalToMultiInstance.html (1.7)
- filters/unsupervised/attribute/RandomSubset.html (1.2)
- filters/unsupervised/attribute/Remove.html (1.9)
- filters/unsupervised/attribute/RemoveType.html (1.8)
- filters/unsupervised/attribute/Reorder.html (1.5)
- filters/unsupervised/attribute/ReplaceMissingValues.html (1.11)
- filters/unsupervised/attribute/Standardize.html (1.12)
- filters/unsupervised/attribute/Wavelet.html (1.4)

2008-04-16 22:11 fracpete

```
introduction of interface RevisionHandler which enables classes to return their CVS/Subversion revision string
```

- classifiers/evaluation/EvaluationUtils.html (1.11)
- classifiers/evaluation/ThresholdCurve.html (1.23)
- classifiers/mi/CitationKNN.html (1.9)
- classifiers/mi/MDD.html (1.4)
- classifiers/mi/MIBoost.html (1.6)
- classifiers/mi/MIDD.html (1.4)
- classifiers/mi/MIEMDD.html (1.6)
- classifiers/mi/MILR.html (1.4)
- classifiers/mi/MINND.html (1.5)
- classifiers/mi/MIOptimalBall.html (1.5)
- classifiers/mi/MISMO.html (1.6)
- classifiers/mi/MISVM.html (1.6)
- classifiers/mi/MIWrapper.html (1.5)
- classifiers/mi/SimpleMI.html (1.6)
- classifiers/mi/TLD.html (1.6)
- classifiers/mi/TLDSimple.html (1.6)
- core/Attribute.html (1.45)
- core/AttributeLocator.html (1.4)
- core/CheckOptionHandler.html (1.13)
- core/ContingencyTables.html (1.8)
- core/EditDistance.html (1.2)
- core/ListOptions.html (1.2)
- core/Memory.html (1.7)
- core/OptionHandlerJavadoc.html (1.8)
- core/Queue.html (1.10)
- core/Range.html (1.18)
- core/SerializationHelper.html (1.2)
- core/Tag.html (1.13)

2008-04-16 22:11 fracpete

```
introduction of interface RevisionHandler which enables classes to return their CVS/Subversion revision string
```

- associations/AprioriItemSet.html (1.6)
- associations/CaRuleGeneration.html (1.4)
- associations/ItemSet.html (1.13)
- associations/LabeledItemSet.html (1.5)
- associations/PriorEstimation.html (1.7)
- associations/RuleGeneration.html (1.4)
- associations/RuleItem.html (1.5)
- classifiers/bayes/net/search/SearchAlgorithm.html (1.9)
- classifiers/functions/VotedPerceptron.html (1.23)
- classifiers/lazy/IB1.html (1.20)
- classifiers/lazy/IBk.html (1.42)
- classifiers/lazy/KStar.html (1.9)
- classifiers/lazy/LBR.html (1.12)
- classifiers/lazy/LWL.html (1.22)
- classifiers/rules/part/C45PruneableDecList.html (1.9)
- classifiers/rules/part/ClassifierDecList.html (1.13)
- classifiers/rules/part/MakeDecList.html (1.17)
- classifiers/rules/part/PruneableDecList.html (1.10)
- experiment/LearningRateResultProducer.html (1.9)
- experiment/PairedCorrectedTTester.html (1.13)
- experiment/PairedTTester.html (1.35)
- experiment/RemoteExperimentEvent.html (1.7)

2008-04-16 22:11 fracpete

```
introduction of interface RevisionHandler which enables classes to return their CVS/Subversion revision string
```

- experiment/CrossValidationResultProducer.html (1.17)
- experiment/DatabaseResultProducer.html (1.18)
- experiment/Experiment.html (1.27)
- experiment/InstanceQuery.html (1.22)
- experiment/OutputZipper.html (1.8)
- experiment/PairedStats.html (1.10)
- experiment/RegressionSplitEvaluator.html (1.25)
- experiment/RemoteExperimentSubTask.html (1.9)
- experiment/ResultMatrix.html (1.9)
- experiment/ResultMatrixGnuPlot.html (1.5)
- experiment/ResultMatrixHTML.html (1.4)
- experiment/ResultMatrixPlainText.html (1.8)
- experiment/ResultMatrixSignificance.html (1.5)
- experiment/TaskStatusInfo.html (1.5)
- experiment/Tester.html (1.4)
- filters/unsupervised/attribute/AddValues.html (1.3)
- filters/unsupervised/attribute/Copy.html (1.7)
- filters/unsupervised/attribute/InterquartileRange.html (1.3)
- filters/unsupervised/attribute/MergeTwoValues.html (1.10)
- filters/unsupervised/attribute/Normalize.html (1.13)
- filters/unsupervised/attribute/NumericCleaner.html (1.2)
- filters/unsupervised/attribute/NumericTransform.html (1.9)
- filters/unsupervised/attribute/Obfuscate.html (1.7)
- filters/unsupervised/attribute/PartitionedMultiFilter.html (1.5)
- filters/unsupervised/attribute/RELAGGS.html (1.3)
- filters/unsupervised/attribute/RandomProjection.html (1.11)
- filters/unsupervised/attribute/RemoveUseless.html (1.10)
- filters/unsupervised/attribute/StringToNominal.html (1.9)
- filters/unsupervised/attribute/SwapValues.html (1.9)
- filters/unsupervised/attribute/TimeSeriesDelta.html (1.7)
- filters/unsupervised/attribute/TimeSeriesTranslate.html (1.9)

2008-04-16 22:11 fracpete

```
introduction of interface RevisionHandler which enables classes to return their CVS/Subversion revision string
```

- core/AllJavadoc.html (1.5)
- core/AttributeExpression.html (1.5)
- core/AttributeStats.html (1.10)
- core/BinarySparseInstance.html (1.13)
- core/ChebyshevDistance.html (1.2)
- core/Check.html (1.2)
- core/CheckScheme.html (1.4)
- core/ClassDiscovery.html (1.7)
- core/Debug.html (1.8)
- core/EuclideanDistance.html (1.13)
- core/FastVector.html (1.16)
- core/FindWithCapabilities.html (1.5)
- core/InstanceComparator.html (1.4)
- core/Javadoc.html (1.6)
- core/Jython.html (1.2)
- core/ManhattanDistance.html (1.2)
- core/Matrix.html (1.25)
- core/Option.html (1.7)
- core/PropertyPath.html (1.2)
- core/ProtectedProperties.html (1.6)
- core/RandomVariates.html (1.5)
- core/SelectedTag.html (1.11)
- core/SerializedObject.html (1.12)
- core/SingleIndex.html (1.8)
- core/SpecialFunctions.html (1.8)
- core/Stopwords.html (1.6)
- core/StringLocator.html (1.4)
- core/SystemInfo.html (1.5)
- core/TechnicalInformation.html (1.10)
- core/TechnicalInformationHandlerJavadoc.html (1.6)
- core/Tee.html (1.4)
- core/TestInstances.html (1.10)
- core/Trie.html (1.2)
- core/Utils.html (1.60)

2008-04-16 22:11 fracpete

```
introduction of interface RevisionHandler which enables classes to return their CVS/Subversion revision string
```

- classifiers/functions/GaussianProcesses.html (1.8)
- classifiers/functions/IsotonicRegression.html (1.7)
- classifiers/functions/LeastMedSq.html (1.17)
- classifiers/functions/LibSVM.html (1.13)
- classifiers/functions/LinearRegression.html (1.24)
- classifiers/functions/MultilayerPerceptron.html (1.12)
- classifiers/functions/PLSClassifier.html (1.4)
- classifiers/functions/PaceRegression.html (1.9)
- classifiers/functions/RBFNetwork.html (1.10)
- classifiers/functions/SMOreg.html (1.14)
- classifiers/functions/SVMreg.html (1.5)
- classifiers/functions/SimpleLinearRegression.html (1.10)
- classifiers/functions/SimpleLogistic.html (1.13)
- classifiers/functions/Winnow.html (1.13)
- classifiers/trees/BFTree.html (1.5)
- classifiers/trees/LMT.html (1.12)
- classifiers/trees/RandomForest.html (1.13)
- classifiers/trees/UserClassifier.html (1.30)

2008-04-16 22:11 fracpete

```
introduction of interface RevisionHandler which enables classes to return their CVS/Subversion revision string
```

- experiment/AveragingResultProducer.html (1.18)
- experiment/CSVResultListener.html (1.13)
- experiment/ClassifierSplitEvaluator.html (1.29)
- experiment/CostSensitiveClassifierSplitEvaluator.html (1.18)
- experiment/DatabaseResultListener.html (1.14)
- experiment/DatabaseUtils.html (1.34)
- experiment/InstancesResultListener.html (1.11)
- experiment/PairedStatsCorrected.html (1.5)
- experiment/PropertyNode.html (1.7)
- experiment/RandomSplitResultProducer.html (1.20)
- experiment/RemoteEngine.html (1.10)
- experiment/RemoteExperiment.html (1.16)
- experiment/ResultMatrixCSV.html (1.4)
- experiment/ResultMatrixLatex.html (1.6)
- experiment/Stats.html (1.12)

2008-04-16 22:11 fracpete

```
branches:  1.1.2;
introduction of interface RevisionHandler which enables classes to return their CVS/Subversion revision string
```

- core/RevisionHandler.html (1.1)

2008-04-16 22:11 fracpete

```
introduction of interface RevisionHandler which enables classes to return their CVS/Subversion revision string
```

- classifiers/trees/ADTree.html (1.8)
- classifiers/trees/Id3.html (1.23)
- classifiers/trees/J48.html (1.9)
- classifiers/trees/M5P.html (1.10)
- classifiers/trees/NBTree.html (1.10)
- classifiers/trees/REPTree.html (1.26)
- classifiers/trees/RandomTree.html (1.20)
- classifiers/trees/SimpleCart.html (1.4)
- core/converters/ArffSaver.html (1.8)
- core/converters/C45Loader.html (1.15)
- core/converters/C45Saver.html (1.7)
- core/converters/CSVLoader.html (1.19)
- core/converters/CSVSaver.html (1.9)
- core/converters/ConverterUtils.html (1.15)
- core/converters/DatabaseConnection.html (1.8)
- core/converters/DatabaseLoader.html (1.14)
- core/converters/DatabaseSaver.html (1.12)
- core/converters/LibSVMLoader.html (1.4)
- core/converters/LibSVMSaver.html (1.4)
- core/converters/Loader.html (1.11)
- core/converters/Saver.html (1.4)
- core/converters/SerializedInstancesLoader.html (1.14)
- core/converters/SerializedInstancesSaver.html (1.7)
- core/converters/TextDirectoryLoader.html (1.4)
- core/converters/XRFFLoader.html (1.4)
- core/converters/XRFFSaver.html (1.5)

2008-04-16 22:11 fracpete

```
introduction of interface RevisionHandler which enables classes to return their CVS/Subversion revision string
```

- classifiers/trees/m5/CorrelationSplitInfo.html (1.4)
- classifiers/trees/m5/RuleNode.html (1.13)
- classifiers/trees/m5/SplitEvaluate.html (1.3)
- classifiers/trees/m5/YongSplitInfo.html (1.4)
- clusterers/forOPTICSAndDBScan/DataObjects/EuclidianDataObject.html (1.5)
- clusterers/forOPTICSAndDBScan/DataObjects/ManhattanDataObject.html (1.5)
- datagenerators/classifiers/regression/Expression.html (1.5)
- datagenerators/classifiers/regression/MexicanHat.html (1.4)
- experiment/xml/XMLExperiment.html (1.6)
- gui/sql/DbUtils.html (1.4)

2008-04-16 22:11 fracpete

```
introduction of interface RevisionHandler which enables classes to return their CVS/Subversion revision string
```

- associations/gsp/Element.html (1.2)
- associations/gsp/Sequence.html (1.2)
- classifiers/bayes/net/search/fixed/FromFile.html (1.8)
- classifiers/bayes/net/search/fixed/NaiveBayes.html (1.6)
- classifiers/functions/neural/NeuralNode.html (1.8)
- classifiers/functions/neural/SigmoidUnit.html (1.7)
- classifiers/meta/ensembleSelection/EnsembleMetricHelper.html (1.2)
- classifiers/meta/ensembleSelection/EnsembleSelectionLibrary.html (1.2)
- classifiers/meta/ensembleSelection/EnsembleSelectionLibraryModel.html (1.2)
- classifiers/meta/ensembleSelection/ModelBag.html (1.2)
- classifiers/trees/m5/Impurity.html (1.8)
- classifiers/trees/m5/PreConstructedLinearModel.html (1.6)
- classifiers/trees/m5/Rule.html (1.15)
- classifiers/trees/m5/Values.html (1.7)
- classifiers/xml/XMLClassifier.html (1.6)
- core/neighboursearch/balltrees/BallNode.html (1.2)
- core/neighboursearch/balltrees/BallSplitter.html (1.2)
- core/neighboursearch/balltrees/BallTreeConstructor.html (1.3)
- core/neighboursearch/balltrees/BottomUpConstructor.html (1.3)
- core/neighboursearch/balltrees/MedianDistanceFromArbitraryPoint.html (1.2)
- core/neighboursearch/balltrees/MedianOfWidestDimension.html (1.2)
- core/neighboursearch/balltrees/MiddleOutConstructor.html (1.3)
- core/neighboursearch/balltrees/PointsClosestToFurthestChildren.html (1.2)
- core/neighboursearch/balltrees/TopDownConstructor.html (1.3)
- core/tokenizers/AlphabeticTokenizer.html (1.2)
- core/tokenizers/CharacterDelimitedTokenizer.html (1.3)
- core/tokenizers/Tokenizer.html (1.3)
- datagenerators/clusterers/BIRCHCluster.html (1.8)
- datagenerators/clusterers/SubspaceCluster.html (1.5)
- datagenerators/clusterers/SubspaceClusterDefinition.html (1.5)
- filters/AllFilter.html (1.13)
- filters/CheckSource.html (1.3)
- filters/Filter.html (1.42)
- filters/MultiFilter.html (1.7)

2008-04-16 02:08 mhall

```
Refactored to convert SubsetEvaluator and AttributeEvaluator from abstract classes to interfaces
```

- attributeSelection/AttributeEvaluator.html (1.11)
- attributeSelection/HoldOutSubsetEvaluator.html (1.7)
- attributeSelection/SubsetEvaluator.html (1.11)
- attributeSelection/UnsupervisedAttributeEvaluator.html (1.10)
- attributeSelection/UnsupervisedSubsetEvaluator.html (1.10)

2008-04-15 02:56 mhall

```
Now safe for a headless environment
```

- gui/beans/DataVisualizerBeanInfo.html (1.3)

2008-04-14 23:08 mhall

```
Now safe for a headless environment
```

- gui/beans/TextViewer.html (1.13)

2008-04-08 03:36 mhall

```
Updated some informational messages
```

- gui/beans/BeanConnection.html (1.8)
- gui/beans/ClassifierPerformanceEvaluator.html (1.19)
- gui/beans/TextViewerBeanInfo.html (1.3)

2008-04-07 04:23 mhall

```
Added support for custom component names
```

- gui/beans/ClustererPerformanceEvaluator.html (1.5)

2008-04-07 04:19 mhall

```
Added support for custom component names
```

- gui/beans/ClassAssigner.html (1.14)
- gui/beans/ClassValuePicker.html (1.8)
- gui/beans/IncrementalClassifierEvaluator.html (1.15)
- gui/beans/MetaBean.html (1.10)

2008-04-07 04:17 mhall

```
Added a couple of methods for getting/setting a custom name for a component
```

- gui/beans/BeanCommon.html (1.4)

2008-04-04 01:52 mhall

```
Added relative path support
```

- gui/beans/SerializedModelSaverCustomizer.html (1.3)

2008-04-03 23:30 mhall

```
Moved relative path stuff to Utils.
```

- gui/experiment/DatasetListPanel.html (1.27)

2008-04-03 22:29 mhall

```
Added support for relative paths for files/directories
```

- gui/beans/LoaderCustomizer.html (1.15)
- gui/beans/SaverCustomizer.html (1.10)

2008-04-03 22:22 mhall

```
Added ability to use relative file/directory paths
```

- core/converters/AbstractFileSaver.html (1.5)
- core/converters/FileSourcedConverter.html (1.5)

2008-03-31 22:12 mhall

```
Fixed null ptr bug introduced by David Zerbetto's changes
```

- gui/beans/KnowledgeFlow.html (1.30)

2008-03-18 03:33 mhall

```
Initial import of Bruno Woltzenlogel Paleo's classes for string-based distance functions.
```

- core/AbstractStringDistanceFunction.html (1.1)

2008-03-15 08:30 fracpete

```
DatabaseUtils now returns ResultSets that are scrollable, if the JDBC driver allows that
SqlViewer works now also with databases that do not return a scrollable ResultSet (like sqlite 3.x or older HSQLDB versions)
corrected key shortcuts of buttons in SqlViewer GUI
fixed Javadoc
```

- experiment/DatabaseUtils.props.sqlite3 (1.2)
- gui/sql/ConnectionPanel.html (1.3)
- gui/sql/QueryPanel.html (1.4)
- gui/sql/ResultSetHelper.html (1.3)
- gui/sql/ResultSetTableModel.html (1.3)
- gui/sql/SqlViewer.html (1.7)
- gui/sql/event/QueryExecuteListener.html (1.2)

2008-03-05 03:23 mhall

```
branches:  1.1.2;
Initial import
```

- gui/beans/SerializedModelSaverBeanInfo.html (1.1)

2008-03-05 03:20 mhall

```
branches:  1.1.2;
Initial import
```

- gui/beans/icons/SerializedModelSaver.gif (1.1)
- gui/beans/icons/SerializedModelSaver\_animated.gif (1.1)

2008-03-05 02:55 mhall

```
Changed the train/test constants to have public rather than private access.
```

- gui/beans/BatchClustererEvent.html (1.4)

2008-02-29 01:26 mhall

```
Added ability to output progress info to the status bar and the option to output per-class info retrieval stats.
```

- gui/beans/IncrementalClassifierEvaluatorBeanInfo.html (1.4)

2008-02-29 01:25 mhall

```
branches:  1.1.2;
Initial import
```

- gui/beans/IncrementalClassifierEvaluatorCustomizer.html (1.1)

2008-02-20 03:09 mhall

```
Made improvements to code for stopping these components when the stop button is pressed in the KnowledgeFlow.
```

- gui/beans/AbstractTrainingSetProducer.html (1.7)

2008-02-20 02:35 mhall

```
Made some methods synchronized
```

- gui/LogPanel.html (1.18)
- gui/WekaTaskMonitor.html (1.9)

2008-02-20 00:38 mhall

```
Made serializable
```

- gui/ExtensionFileFilter.html (1.8)

2008-02-18 02:20 mhall

```
Fixed bug where if the window was closed (rather than ok or cancel being pressed) the connection would be made. Closing the window is now the same as canceling.
```

- gui/DatabaseConnectionDialog.html (1.8)

2008-02-18 02:17 mhall

```
Fixed a couple of bugs where database connection was not being closed if close/cancel was pressed. Thanks to Miryam Gomez and Raquel Porras for the bug fix
```

- gui/experiment/ResultsPanel.html (1.44)

2008-01-31 01:59 mhall

```
Small addition about double-click support.
```

- gui/beans/README\_KnowledgeFlow (1.6)

2008-01-29 21:00 mhall

```
Changed so that percentage split now uses double rather than int for expressing the percentage.
```

- gui/explorer/ClassifierPanel.html (1.111)

2008-01-18 04:21 fracpete

```
removed proprietary com.sun.image.codec.jpeg.JPEGCodec etc classes and replaced them with javax.imageio ones
```

- gui/boundaryvisualizer/BoundaryPanel.html (1.23)
- gui/visualize/JPEGWriter.html (1.6)

2008-01-11 02:33 fracpete

```
added memory usage frame for keeping an eye on Weka and where the memory gets eaten up
can be configured via the "weka/gui/MemoryUsage.props" props file
```

- gui/GUIChooser.html (1.34)
- gui/Main.html (1.14)
- gui/visualize/VisualizeUtils.html (1.9)

2008-01-11 02:33 fracpete

```
branches:  1.1.2;
added memory usage frame for keeping an eye on Weka and where the memory gets eaten up
can be configured via the "weka/gui/MemoryUsage.props" props file
```

- gui/MemoryUsage.props (1.1)
- gui/MemoryUsagePanel.html (1.1)

2008-01-08 03:00 mhall

```
branches:  1.1.2;
Initial import
```

- gui/beans/Startable.html (1.1)

2007-12-19 00:01 mhall

```
Updated to 3-5-7
```

- core/version.txt (1.6)
